# Dissolution Assay of Bupropion/Naltrexone Hydrochloride Salts of Bilayer Composition Tablets Following the Development and Validation of a Novel HPLC Method

**DOI:** 10.3390/ma15238451

**Published:** 2022-11-27

**Authors:** Anna Apostolidi, Chrystalla Protopapa, Angeliki Siamidi, Marilena Vlachou, Yannis Dotsikas

**Affiliations:** 1 Laboratory of Pharmaceutical Analysis, School of Health Sciences, Department of Pharmacy, National and Kapodistrian University of Athens, 15784 Athens, Greece; 2 Section of Pharmaceutical Technology, School of Health Sciences, Department of Pharmacy, National and Kapodistrian University of Athens, 15784 Athens, Greece

**Keywords:** naltrexone, bupropion, HPLC method, experimental design, dissolution, bilayer tablets

## Abstract

Compounded medicinal products containing bupropion hydrochloride (BUP·HCl) and naltrexone hydrochloride (NTX·HCl) are available as adjunct therapy for the management of weight in obese/overweight adults. The present work describes the development and validation of a novel RP-HPLC method for a simultaneous quantitation during the dissolution of both drugs from compounded bilayer composition tablets. The method involves a Nucleosil 100-3 C-18 column (4.6 × 150 mm) and a mobile phase of a 70%/30% *v*/*v* ACN/KH_2_PO_4_·H_2_O aqueous solution of a 5 mM concentration. The flow rate was set at 1.35 mL/min and the detection was conducted using UV spectrophotometry (λ_max_ 214 nm). The method was validated according to the ICH guidelines and fulfilled the specifications for the specificity, linearity, accuracy, precision and stability for both the sample and standard solutions. Furthermore, the robustness of the method was evaluated by applying a fractional factorial experimental design and by utilizing both graphical and statistical approaches to identify the HPLC factors that should be strictly controlled during the analysis. The method proved to be suitable for the analysis of the dissolution samples and, consequently, the release of BUP·HCl and NTX·HCl from the formulations.

## 1. Introduction

The metabolic syndrome and obesity are two major health issues affecting over two billion adults worldwide, according to the World Health Organization (WHO). It is estimated that, by 2025, over 2.7 billion people are going to be overweight, with 1.7 billion of those severely affected by obesity [1]. Obesity is also an emerging health issue for children and teenagers; over 120 million adolescents aged 5–19 years old were obese in 2016 [2]. The burden of this condition does not just refer to physical well-being. It affects mental health, social activity and the financial situation not only of the patient, but of the health care system too. In less than a decade, from 1998 to 2006, obesity was responsible for an increase of USD 40 billion in medical expenses in the USA [3]. Obesity is linked to a variety of diseases, including type II diabetes, cardiovascular problems, such as hypertension and atherosclerosis, stroke and certain types of cancer. All these are a few of the leading causes of preventable and premature death [4].

Over the past 70 years, several drugs have been approved for the management of obesity. The majority of these medications target the central neural system, but the side effects that have been reported, combined with a potentially harmful profile, have led to their withdrawal. Modern therapies have been developed, and even though they also have central mechanisms of action, they are relatively safe and have been licensed by the authorities [5].

**Figure 1:**
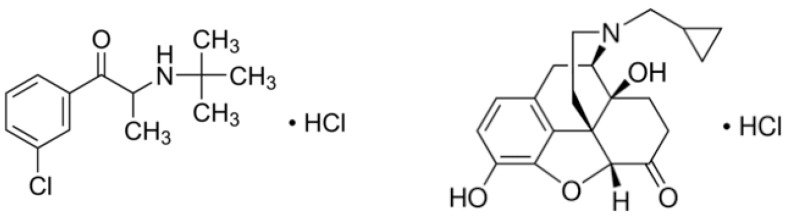
Chemical structures of racemic bupropion hydrochloride (**left**) and naltrexone hydrochloride (**right**).

The combination of bupropion and naltrexone is one of those formulations used in the long-term management of obesity and overweight, in addition to a reduced-calorie diet and exercise. Both of these active substances (APIs) have alone and in combination shown to have potential, with the synergistic combination being more beneficial. The exact mechanism of action of this combination is not fully understood. It seems to suppress the appetite, affecting two principal areas of the brain: the arcuate nucleus of the hypothalamus mesolimbic dopaminergic reward system [6]. The two active ingredients, naltrexone and bupropion, act on the parts of the brain that control food intake and energy balance, while limiting the activity of the part of the brain that controls the enjoyment of food consumption. Coadministered, they reduce the appetite, and, consequently, the amount of food that patients consume, and increase energy consumption, helping patients to follow a diet of a controlled caloric intake and helping reduce their weight [7]. The bilayer tablets, which consist of monolithic partially coated or multilayered matrices, are extensively used for the achievement of a controlled and sequential release of two separate drugs when they are incompatible or when one of the drugs needs to be released immediately and the other at a controlled rate. In the case of the compounded NTX·HCl–BUP·HCl (Figure 1), a bilayer tablet is needed, since the concentration difference between the two drugs is noticeably different, so that a modified release for both drugs is achieved [8,9,10,11].

Only two HPLC methods have been found for the determination of these two APIs in formulations [12,13], but, thus far, there is no validated HPLC–UV method for the simultaneous quantitation of bupropion and naltrexone during their dissolution studies. In view of this, it was intriguing to develop and validate a novel HPLC method with improved characteristics specific for the analysis of the dissolution samples. The method was, indeed, validated according to the current ICH guidelines. Regarding the robustness of this method, an experimental design was employed to clarify the parameters with potential significant effects on the chromatographic responses. Lastly, the current method was applied to the estimation of the release profile of two novel formulations of bilayer composition tablets containing both APIs.

## 2. Materials and Methods

### 2.1. Reagents and Solvents

BUP·HCl (MW: 276.20, pKa = 8.22, >98.0% purity) and NTX·HCl (MW: 377.87 g/mol, pKa = 9.35, >98.0% purity) were purchased from Tokyo Chemical Industry (Tokyo, Japan). HPMC K15M and PEO (MW: 7 × 10^6^ g/mol) were purchased from Sigma-Aldrich (Steinheim, Germany). Eudragit^®^ L100-55 was obtained from Rohm GmbH Pharma Polymers (Darmstadt, Germany). Magnesium stearate was supplied by Riedel-De Haen (Hannover, Germany). HPLC-grade methanol, acetonitrile, potassium dihydrogen phosphate monohydrate and hydrochloric acid (1 M), were obtained from Sigma-Aldrich (Steinheim, Germany). HPLC-grade water was obtained from a Millipore Milli-Q device (Merck S.A. Hellas, Athens, Greece).

### 2.2. Chromatographic Conditions

HPLC experiments were performed using a GBC system, including an LC 1120 isocratic pump and a GBC LC 1210 UV–Vis detector (Darmstadt, Germany). All samples were introduced via a Rheodyne injector valve with a sample loop of 20 μL. Empower 2 software was employed for data acquisition and analysis (Waters, Milford, MA, USA).

The mobile phase consisted of a mixture of ACN and 5 mM of KH_2_PO_4_^·^H_2_O at a ratio of 70%/30% *v*/*v* under isocratic conditions. Chromatographic separations were performed in a Nucleosil 100-3 C-18 column (4.6 × 150 mm) with the flow rate set at 1.35 mL/min, the detection wavelength at 214 nm and the injection volume at 20 μL. All mobile phases during the development of the method and its validation were passed through a 0.45 μm nylon-membrane filter, which was purchased from Gelman Sciences (Northampton, UK), and degassed prior to use.

### 2.3. Preparation of the BUP·HCl and NTX·HCl Modified-Release Matrix Tablets

Each API was blended with the excipients HPMC K15, Eudragit L100-55, PEO and Mg-stearate using a laboratory-scale powder blender at 32 rpm for 10 min. The lubricant (magnesium stearate) was then added to each of the APIs, after which mixing was continued for 5 more minutes. The BUP·HCl powder mixture (200 mg) (Table 1) was loaded first onto a 10 mm diameter dye and then tamped. The addition of the NTX·HCl mixture (130 mg) was then followed and both powders were, subsequently, directly compressed in a hydraulic press (Maassen type, MP 150) under a loading compression force of 5.00–5.50 tons.

### 2.4. Postcompression Parameters

Thickness test: The tablets’ thickness was determined using a Vernier caliper scale [14].

Hardness test: The hardness of the tablets was measured on a Erweka hardness tester (Erweka, typeTBH28). The force exerted was the same to that used for breaking the tablet in a diametric compression. The surface hardness was measured in N [14].

### 2.5. In Vitro Dissolution Studies

The dissolution tests of the prepared tablets were performed in a test apparatus USP type II (Pharmatest, Hainerp, Germany) (paddle method, 50 rpm) in two different aqueous media; the dissolution profile of BUP·HCl and NTX·HCl was monitored in two steps by withdrawing solutions of the samples (5 mL) every 30 min for the first 2 h from a 450 mL 1.2 pH HCl solution (0.063 M) to simulate the gastric environment and every hour for the next 6 h from a 6.8 pH aqueous medium to simulate the intestinal fluids. The 6.8 pH medium was created by adding another 450 mL of a buffer K_2_HPO_4_ solution to the 450 mL 1.2 pH solution (0.14 M, pH 9.0), reaching a final volume of 900 mL (2); the withdrawn volume of the aqueous media was replenished. The temperature of the dissolution media was kept stable at 37 ± 0.5 °C.

Samples from the dissolution studies were diluted using two different methods depending on the pH of the medium in order to have the same chromatographic profile. Specifically, regarding the samples collected from 30 min to 120 min, where the pH was set to 1.2, 500 μL of the dissolution test sample was initially diluted with 500 μL of MeOH, and then further diluted with a mixture of MeOH/HCl 0.2 M 50%:50% *v*/*v* till the volume of 2 mL was reached. Samples withdrawn from 180 to 480 min (500 μL) were diluted to a final volume of 2 mL with 1.5 mL of a MeOH/HCl 0.2 M 50%:50% *v*/*v* mixture. Each sample was injected into the HPLC apparatus following vortex mixing.

### 2.6. Preparation of Stock and Working Solutions

In order to prepare the stock solutions, the respective amounts of BUP·HCl and NTX·HCl were accurately weighed and dissolved in methanol into a 50 mL volumetric flask, thus, obtaining a concentration of 100 μg/mL for both APIs. Thereafter, the appropriate dilutions were created from each stock solution with MeOH/HCl 0.2 M 50%:50% *v*/*v*, leading to the desired working standard solutions. All solutions were kept in a refrigerator.

### 2.7. Solution for Selectivity Estimation

A placebo mixture of the excipients of each formulation was prepared in the concentration ratio corresponding to the content in the tablets. A sample solution mixture containing NTX·HCl and BUP·HCl at the concentrations corresponding to their specification limits (5 and 45 μg/mL, respectively) was used to prove the method’s selectivity.

### 2.8. Solutions for the Estimation of Linearity

For this purpose, five standard solutions were analyzed, each in triplicate, covering the range of 20–160% of the nominal concentrations for NTX·HCl (5 μg/mL) and the range of 8.88–122.2% for BUP·HCl (45 μg/mL). In detail, the drug concentrations for NTX·HCl were 1.0, 2.0, 4.0, 6.0 and 8.0 μg/mL, whereas for BUP·HCl, the relevant concentrations were 4.0, 8.0, 16.0, 32.0 and 55.0 μg/mL.

### 2.9. Solutions for the Estimation of Accuracy

The accuracy was assessed in terms of the % recovery through spiking, in triplicate, each placebo mixture with known amounts of both APIs in order to obtain concentrations corresponding to 80, 100 and 120% concentration levels, respectively. More specifically, the concentrations used were 36 (80%)–45 (100%)–54 (120%) μg/mL for BUP·HCl and 4 (80%)–5 (100%)–6 (120%) μg/mL for NTX·HCl.

### 2.10. Solutions for the Estimation of Precision

In the current study, repeatability and intermediate precision were estimated using six replicate samples spiked at a concentration of 100% for both APIs (5 μg/mL for NTX·HCl and 45 μg/mL for BUP·HCl). The solutions were injected into the HPLC instrument the same day (repeatability) and over three consecutive days (intermediate precision). The retention time and the peak area were determined and the results were expressed through a % RSD of time and area. The reproducibility was not assessed, as the method would not be transferred to an external laboratory.

### 2.11. Stability Study

The stability of the sample solutions was estimated for a 2-day period (0 h, 4 h, 24 h and 48 h) under two different conditions (refrigerated and environmental storage). Six independent solutions of 100% concentration for both APIs were prepared, three of which were stored in the refrigerator (4 °C), whereas the other three were stored at room temperature (25 °C) in a cupboard, away from direct sunlight exposure.

### 2.12. Robustness Testing

Robustness evaluated the ability of the method to remain unaffected through random changes of its parameters, and it was used to show the reliability of the method. It was performed by deliberately changing the chromatographic conditions, often using an experimental design, and was required for a full method validation [15]. This way, we could identify factors that had a significant effect on the responses and set the ranges of their variation. In the present work, we chose to apply a fractional factorial design (2^5−1^), with the factors under investigation being (A) the % ACN content, (B) the flow rate of the mobile phase, (C) the detection wavelength, (D) the salt concentration (mM) and (E) the HCl concentration (M). Each factor was tested in two levels (−1, +1) with small changes from the nominal value (0). A solution containing NTX·HCl and BUP·HCl, at the concentrations corresponding to their specification limits (5 and 45 μg/mL, respectively), was injected into the HPLC in all experiments for the determination of robustness. Table 2 presents the experimental factors and their examined levels, as suggested by Design-Expert^®^ software v.13 trial version (Stat-Ease-Inc., Minneapolis, MN, USA).

Table 3 presents the 19 runs conducted for the fractional factorial design with three additional points (replicates of the central values). The following responses were evaluated: the retention times (t_R_), the areas, the number of theoretical plates (N), the tailing factors (T_f_) for both APIs and the resolution between them.

## 3. Results and Discussion

### 3.1. Method Development

Based on the physiochemical properties of both analytes, a reverse-phase HPLC column was chosen. Both the C-8 and C-18 columns were tested during the method’s development using various mixtures of MeOH/H_2_O and ACN/H_2_O, including salts at different concentrations. In most cases the retention times of BUP·HCl were >20 min, and either NTX·HCl exhibited a poor retention (overlap with dead time) or the peaks were overlapping. Peak symmetry was also an issue in most trials. After considerable experimentation with a number of columns, we concluded that a C-18 column (CC 150/4.6 Nucleosil 100-3 C18), a mobile phase consisting of ACN and 5 mM of KH_2_PO_4_ at a ratio of 70%/30% *v*/*v*, under isocratic conditions and a flow rate of 1.35 mL/min were proved optimal for the target sought. Based on relevant bibliographic UV spectral data [16] and trials at the laboratory, the wavelength was set at 214 nm.

### 3.2. Validation

Following the development of the method, its validation was investigated in accordance with the ICH guidelines in order to prove its suitability [17]. The parameters that were evaluated were specificity, linearity, limit of detection (LOD), limit of quantitation (LOQ), accuracy, precision and stability.

#### 3.2.1. Specificity

**Figure 2:**
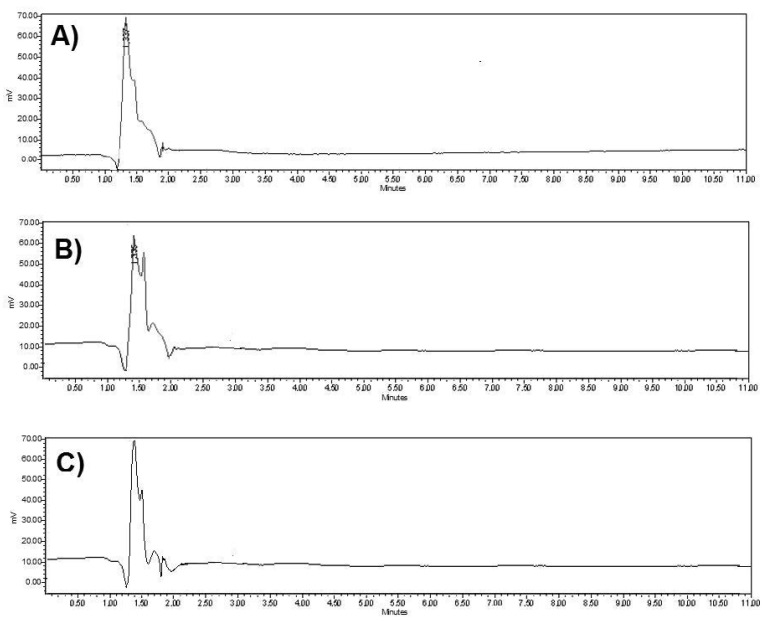
Representative chromatograms of (**A**) diluent, (**B**) placebo of formulation A and (**C**) placebo of formulation B.

**Figure 3:**
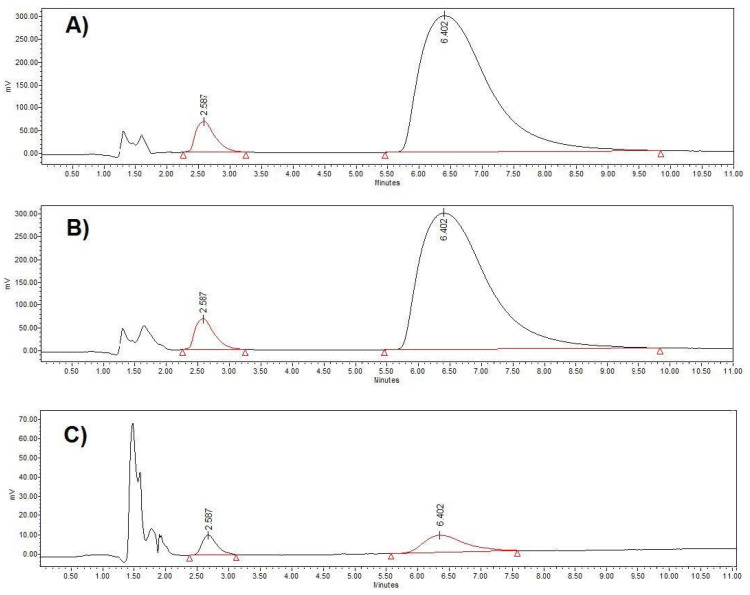
Representative chromatograms of (**A**) standard sample, (**B**) sample obtained from the dissolution study of formulation A from the 6.8 pH medium and (**C**) sample obtained from the dissolution study of formulation B from the 1.2 pH medium. NTX·HCl was eluted at 2.56 min and BUP·HCl was eluted at 6.40 min.

The evaluation of specificity revealed that both APIs could be identified and determined with no interferences from the diluent or the excipients. More specifically, the placebo solution, prepared from excipients at the appropriate ratios, had no influence on the chromatographic profile (Figure 2 and Figure 3). Furthermore, the two peaks showed acceptable values of resolution, Rs >> 2, as NTX·HCl was eluted at 2.56 min, whilst BUP·HCl was eluted at 6.40 min (Figure 3).

#### 3.2.2. Linearity, LOD and LOQ

Two calibration curves were constructed following the analysis of five standard samples of BUP·HCl and NTX·HCl, each one in triplicate.

The R^2^ values were greater than 0.9996, suggesting a strong linear correlation between the areas (y) and the concentrations (x) for each API. Moreover, the LOD and LOQ values were calculated from calibration curves, as depicted in Table 4.

#### 3.2.3. Accuracy

According to the ICH, the criteria for the mean % recovery of APIs in medicinal products are 98% ≤ %R ≤ 102%. In the current study, these criteria were met for all levels (80%, 100% and 120%) for both recipes (Table 5).

#### 3.2.4. Precision

Samples at 100% concentration were prepared in order to evaluate the repeatability and intermediate precision (IP). The criterion of the % RSD ≤ 2 was met, proving the method to be precise. Table 6 presents the % of the relative error, as calculated from the calibration curve for the six replicate samples, as well as the % RSD. Regarding the IP, the % RSDR values were 0.78 and 0.62 for BUP·HCl and NTX·HCl, respectively.

#### 3.2.5. Stability

After performing the stability tests, it was concluded that both standard and sample solutions had no stability issues during a 24 h period, both under environmental and refrigerated conditions, and this period was more than adequate for the analysis of all samples. For a 48 h period, this conclusion was not valid, as a % of deviation of >5% was observed for both APIs.

### 3.3. Robustness Results

The results of the 19 experiments for robustness testing are presented in Table 7. The following responses were estimated: the retention times (t_R_), the areas, the number of theoretical plates (N), the tailing factors (T_f_) and the resolution (R) [18] between the two analytes.

For the evaluation of the significant effects, the graphical approach was implemented [19,20,21]. The normal and half-normal plot graphs, along with Pareto charts, were utilized for this purpose. The first two plots led to the same conclusion, i.e., the factors that were off the red line had a significant effect on the area responses. The difference between the two plots was that the half-normal plot did not indicate a positive or negative effect of the factor, whereas the normal plot did, as it depicted on which side of the line the factor was. The Design-Expert^®^ software indicated the effect (positive or negative) of the factor (vide color code: orange for positive, blue for negative). The half-normal plots could help indicate the size of the effect, because all the factors were on the same side of the red line.

**Figure 4:**
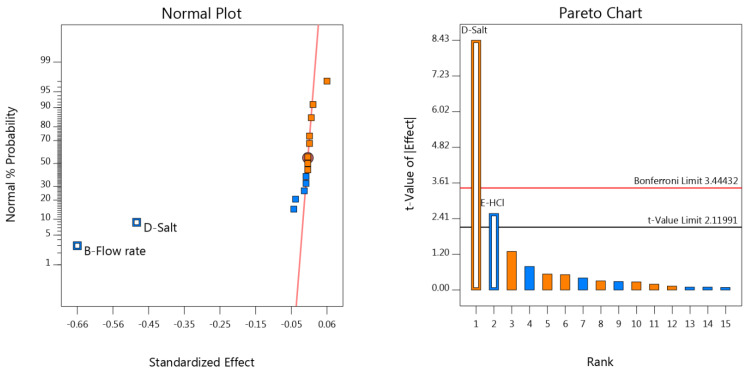
Representative normal plot graph for the response of NTX·HCl tR (**left**) and Pareto chart for the response of BUP·HCl (N) (**right**). A: %ACN; B: flow rate (mL/min); C: wavelength (nm); D: salt concentration (mM); E: HCl concentration (M).

The Pareto chart was also utilized. It included the Bonferroni and the *t*-value limits. Factors that exceeded the Bonferroni limit were considered to have a significant effect on the selected response, while those that did not exceed the *t*-value limit were considered non-significant. Lastly, the factors with bars between these two limits were considered potentially significant, and should be further examined (Figure 4).

As seen in the graphs and charts for all responses, the number of factors that were significant for each response was limited, and this was an indication of the method being robust. Specifically, for the retention times of both analytes, the flow rate and salt concentration were proven to be significant. Regarding the areas, flow rate and wavelength, these were found to be significant for NTX·HCl, while the Pareto chart revealed that all factors were below the *t*-value limit in the BUP·HCl area. The concentrations of salt and HCl were estimated as being significant for the theoretical plates of both analytes. The resolution was significantly affected only by the changes in the HCl concentration. Finally, the tailing factor of the NTX·HCl salt concentration was proven to be significant, while no significant factor was found for the tailing factor of BUP·HCl.

### 3.4. Postcompression Parameters

The physical properties of the new formulations (thickness and hardness tests) were, in all cases, within the requisite limits. The thickness data (*n* = 10) showed uniformity in all cases (0.345 ± 0.001 cm for formulation A and 0.345 ± 0.001 for formulation B). The hardness test results (*n* = 10) indicated a uniformity and robustness of the formulations (125.4 ± 0.52 N for formulation A and 126.3 ± 0.67 for formulation B).

### 3.5. Dissolution Study Results

**Figure 5:**
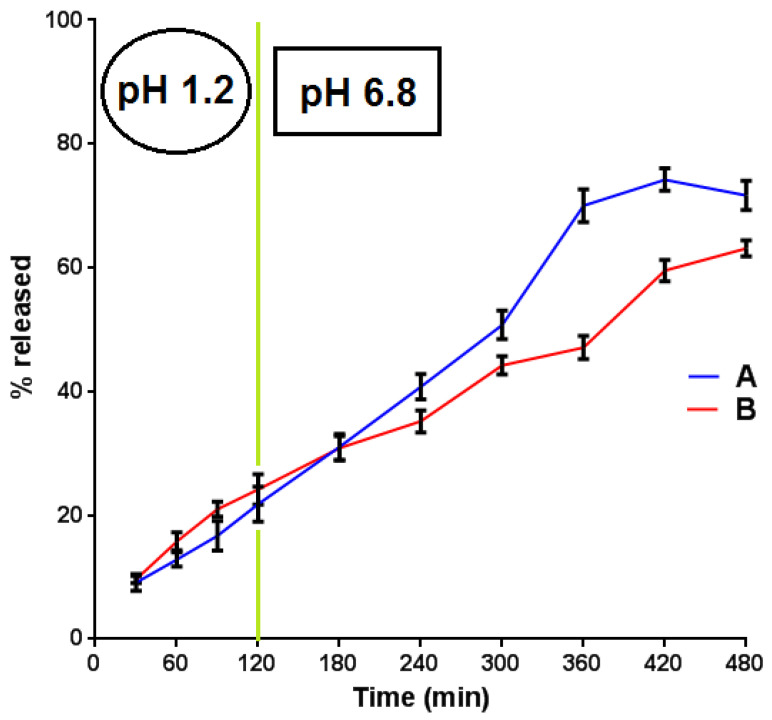
In vitro % release (mean values, N = 3) of BUP·HCl from formulations A and B vs. time.

**Figure 6:**
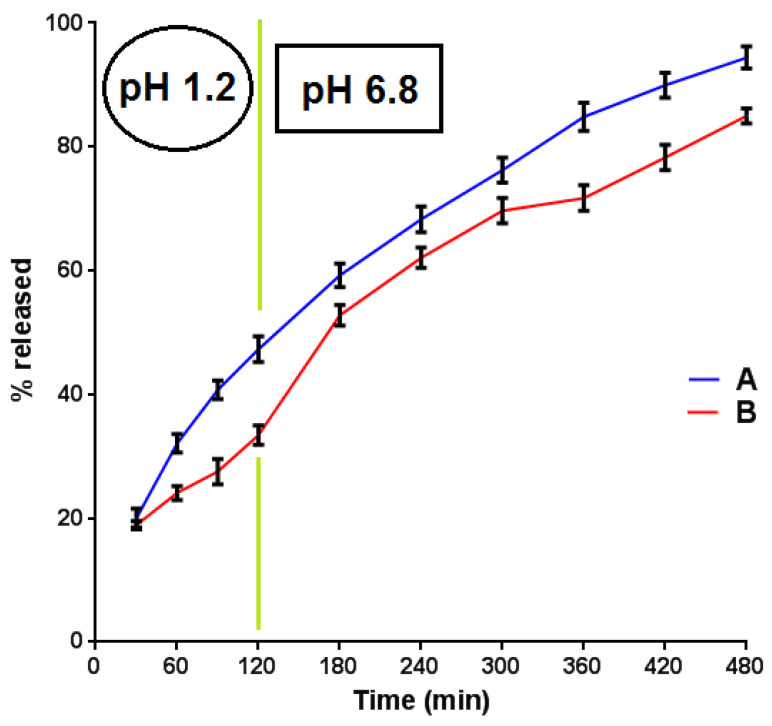
In vitro % release (mean values, N = 3) of NTX·HCl from formulations A and B vs. time.

The new method was employed for the evaluation of the release profile of two novel tablet formulations (A and B). The dissolution results for both APIs are depicted in Figure 5 and Figure 6.

From Figure 5, it became evident that the release of BUP·HCl reached almost 75% from formulation A and 60% from formulation B at t = 420 min. Moreover, the release of BUP·HCl from both A and B followed the requisite pattern (modified release). An analogous-release profile from both the A and B formulations was followed by the NTX·HCl active substance (Figure 6). The fact that the % release of NTX·HCl was in all cases higher than that of BUP·HCl could be attributed to the lower aqueous solubility of the latter, both as a free base (t > 120 min) and as a hydrochloride salt (t < 120 min). Moreover, naltrexone is a five-bond count H-bond acceptor, whilst bupropion a two-bond count H-bond acceptor. This resulted in a more favorable interaction between the water and naltrexone than between the water molecules and bupropion, leading to an enhanced solubility and, therefore, a % release of the former.

## 4. Conclusions

A simple and rapid HPLC method was developed and validated for the simultaneous determination of naltrexone hydrochloride and bupropion hydrochloride in dissolution test samples from compounded NTX·HCl–BUP·HCl bilayer tablets. Bilayer tablets are well tolerated by patients and useful for either the sustained and immediate release of the same drug or for the sequential release of two drugs in combination. The combination of NTX·HCl–BUP·HCl in the bilayer tablets led to the sought modified release of both drugs at a different pace and with no interaction between the two molecules. The materials used were inexpensive and readily available, the total run time was just 12 min and the samples did not need any demanding pretreatment. Another advantage of the method was the isocratic conditions employed, thus, avoiding the repeated equilibration of the system, a process which is, inevitably, time consuming. Moreover, the method was found to be linear, specific, precise and accurate, and the samples were stable for 24 h and complied with the ICH guidelines for validation. Thus, the new method is expected to greatly aid the design of in vivo experiments in the future, utilizing compounded NTX·HCl–BUP·HCl formulations.

## Figures and Tables

**Table 1:** The composition of BUP·HCl and NTX·HCl tablet formulation (mg).

Ingredients	Formulation A		Formulation B	
BUP·HCl	-	90	-	90
NTX·HCl	8	-	8	-
HPMC K15M	20	10	15	15
Eudragit L100-55	62	50	45	58
PEO	38	48	60	35
Mg Stearate	2	2	2	2
**Total**	**130**	**200**	**130**	**200**

**Table 2:** Experimental factors and their examined levels.

Experimental Parameters	−1 Level	0 Level (Nominal Value)	+1 Level
**% ACN**	29.0	30.0	31.0
**Flow rate (mL/min)**	1.20	1.35	1.50
**Detection wavelength (nm)**	212.0	214.0	216.0
**Salt concentration (mM)**	4.0	5.0	6.0
**HCl concentration (M)**	0.15	0.20	0.25

**Table 3:** The experiments proposed by the software for the fractional factorial design.

RUN	Factor 1 A: % ACN	Factor 2 B: Flow Rate (mL/min)	Factor 3 C: Wavelength (nm)	Factor 4 D: Salt Concentration (mM)	Factor 5 E: HCl Concentration (M)
1	31.0	1.20	216.0	6.0	0.15
2	29.0	1.20	212.0	6.0	0.15
3	29.0	1.20	216.0	6.0	0.25
4	29.0	1.50	216.0	4.0	0.25
5	30.0	1.35	214.0	5.0	0.20
6	30.0	1.35	214.0	5.0	0.20
7	31.0	1.50	212.0	6.0	0.15
8	31.0	1.20	216.0	4.0	0.25
9	30.0	1.35	214.0	5.0	0.20
10	31.0	1.50	212.0	4.0	0.25
11	29.0	1.50	212.0	6.0	0.25
12	29.0	1.20	212.0	4.0	0.25
13	29.0	1.50	212.0	4.0	0.15
14	31.0	1.20	212.0	6.0	0.25
15	31.0	1.50	216.0	4.0	0.15
16	29.0	1.50	216.0	6.0	0.15
17	31.0	1.20	212.0	4.0	0.15
18	31.0	1.50	216.0	6.0	0.25
19	29.0	1.20	216.0	4.0	0.15

**Table 4:** Regression analysis, LOD and LOQ values.

Analyte	Calibration Curve	R2	LOD (μg/mL)	LOQ (μg/mL)
**NTX·HCl**	y = 282763x − 8232.6	0.9996	0.167	0.500
**BUP·HCl**	y = 529512x − 311738	0.99990	0.333	1.00

**Table 5:** Accuracy results (mean values) obtained for three concentration levels for both formulations.

API	80% Level (Recipe A)	80% Level (Recipe B)	100% Level (Recipe A)	100% Level (Recipe B)	120% Level (Recipe A)	120% Level (Recipe B)
**NTX·HCl**	−1.91	−1.84	−0.58	1.78	−1.49	−1.43
**BUP·HCl**	−1.88	−1.68	−1.47	0.42	−1.92	−0.74

**Table 6:** Repeatability results for BUP·HCl (up) and NTX·HCl (down).

**BUP·HCl**			
	**Theoretical Concentration**	**Calculated Concentration**	**% Error**
**1**	45.0	44.8	−0.36
**2**	45.0	45.3	0.75
**3**	45.0	45.5	1.0
**4**	45.0	44.7	−0.78
**5**	45.0	44.9	−0.10
**6**	45.0	44.2	−1.7
**Average**		44.9	−0.19
**SD**		0.42	
**% RSD**		0.92	
**NTX·HCl**			
	**Theoretical Concentration**	**Calculated Concentration**	**% Error**
**1**	5.00	5.02	0.35
**2**	5.00	4.98	−0.45
**3**	5.00	5.03	0.68
**4**	5.00	5.11	2.15
**5**	5.00	5.04	0.91
**6**	5.00	5.02	0.46
**Average**		5.03	0.68
**SD**		0.039	
**% RSD**		0.77	

**Table 7:** Data of the responses obtained from the experiments of the experimental design.

Run	NTX·HCl t_R_ (min)	BUP·HCl t_R_ (min)	NTX·HCl Area	BUP·HCl Area	NTX·HCl (N)	BUP·HCl (N)	R	NTX·HCl T_f_	BUP·HCl T_f_
**1**	2.90	7.07	1371295	26477780	549	262	3.72	1.61	2.05
**2**	2.92	7.41	2142495	29486116	518	231	3.34	1.71	2.23
**3**	2.89	7.28	1336954	36898090	457	198	3.38	1.75	2.17
**4**	2.67	7.18	1157601	28447033	279	160	3.11	1.48	2.03
**5**	2.71	7.01	1477239	22031048	687	366	4.60	1.24	1.80
**6**	2.71	7.01	1492210	21880682	683	367	4.58	1.26	1.80
**7**	2.30	5.61	1710613	23176233	528	260	3.71	1.56	2.07
**8**	3.44	9.91	1400845	36296497	269	156	3.26	1.46	2.05
**9**	2.68	6.58	1493017	236430000	496	267	3.73	1.12	1.79
**10**	2.74	7.89	1549601	26844224	335	175	3.458	1.52	2.00
**11**	2.29	5.76	1745586	31644700	513	197	3.38	1.67	2.15
**12**	3.38	9.12	2235361	38979921	273	163	3.13	1.49	1.99
**13**	2.73	7.33	1794292	23088739	365	194	3.45	1.46	2.09
**14**	2.87	6.94	2243253	40125975	462	217	3.40	1.65	2.11
**15**	2.76	7.96	1096673	20411993	362	190	3.60	1.46	2.10
**16**	2.31	5.87	950839	21054770	557	229	3.68	1.86	2.17
**17**	3.49	10.06	2293433	29003871	334	192	3.62	1.49	2.21
**18**	2.27	5.48	1090610	29189979	498	219	3.44	1.62	2.09
**19**	3.45	9.33	1477117	26828068	340	197	3.46	1.27	2.11

## Data Availability

Data presented in this study are available on request from the corresponding authors.
